# Distillation of an End-to-End Oracle for Face Verification and Recognition Sensors †

**DOI:** 10.3390/s20051369

**Published:** 2020-03-02

**Authors:** Francesco Guzzi, Luca De Bortoli, Romina Soledad Molina, Stefano Marsi, Sergio Carrato, Giovanni Ramponi

**Affiliations:** 1 Engineering and Architecture department, Image Processing Laboratory (IPL), University of Trieste, 34127 Trieste, Italy; 2 Elettra Sincrotrone Trieste, Scientific Computing, 34149 Basovizza, Italy; 3 The Abdus Salam International Centre for Theoretical Physics (ICTP), Multidisciplinary Laboratory, 34151 Trieste, Italy

**Keywords:** face recognition, face verification, biometric sensors, deep learning, distillation, convolutional neural networks, spatial transformer network

## Abstract

Face recognition functions are today exploited through biometric sensors in many applications, from extended security systems to inclusion devices; deep neural network methods are reaching in this field stunning performances. The main limitation of the deep learning approach is an inconvenient relation between the accuracy of the results and the needed computing power. When a personal device is employed, in particular, many algorithms require a cloud computing approach to achieve the expected performances; other algorithms adopt models that are simple by design. A third viable option consists of model (oracle) distillation. This is the most intriguing among the compression techniques since it permits to devise of the minimal structure that will enforce the same I/O relation as the original model. In this paper, a distillation technique is applied to a complex model, enabling the introduction of fast state-of-the-art recognition capabilities on a low-end hardware face recognition sensor module. Two distilled models are presented in this contribution: the former can be directly used in place of the original oracle, while the latter incarnates better the end-to-end approach, removing the need for a separate alignment procedure. The presented biometric systems are examined on the two problems of face verification and face recognition in an open set by using well-agreed training/testing methodologies and datasets.

## 1. Introduction

### 1.1. Face Recognition Sensors

Face recognition systems represent now a pervasive reality. Smartphones, computers and social networks provide verification, similarity and recognition functions for both security and entertainment purposes. The basic hardware setup exploits a webcam and a single-board PC, that are already used in the device; a separate ‘face sensor module’ may be included to perform face recognition functions. Especially due to the latter class of sensors, many small devices emerged whose output is an identity or a biometric signature. Indeed, they are sold as biometric sensors on distributors’ websites.

Face recognition has pushed more than any other research topic on Convolutional Neural Networks (CNNs) because the impact of human-like performances in this type of Artificial Intelligence is huge [1]. Making a self-learning system understand where and what to observe for reliable identification and the use of biomimicry for the design of the trainable structures provided the biggest performance boost with respect to the results of the previous hand-crafted algorithm.

Neural networks for face recognition are typically trained by the use of a supervised training procedure, in which the right answer to the problem is provided for each input; during the training, the model learns to give the correct answer analyzing the distribution of each input (the pixels) and then, through error back-propagation, enforcing a set of parameters (the weights of convolutional and fully-connected layers). Even if the aforementioned biomimicry has inspired a particular structure and hierarchy for signal processing, presently it is impossible to determine detailed reasons why a certain weight takes a certain value. The same happens for the meaning of each axis in the multi-dimensional output space in which a particular identity is defined. The main point of the current deep learning methods is to avoid defining handcrafted methods that are fully understood, but provide inevitably lower performances. That is why the term “oracle” is used in the literature. In this paper, we use oracle as a synonym of “trained model”.

The common thinking is that the problem of face recognition is completely solved, but in fact, it is not. Present-day systems can be used in consumer products and for a small database of users, but they are unable to provide the high accuracy desired e.g., for a banking system. Until recently, ‘the complex-the better’ paradigm has been the only viable solution, leading to complex and very deep oracles that can operate on a High-Performance Computing (HPC) platform only. For example in [2] a multi-feature fusion algorithm is proposed, but its applicability is constrained to CUDA capable devices. The trend of the latest research [3] follows a complete end-to-end approach (fully neural detection and classification in the same structure), including accurate but complex object detectors (RCNN [4], FCN [5], MMod [6], MTCNN [7]), but unfortunately leading to slow models, even on high-end GPU [8]. For personal devices, instead, two options are available: to accept an inevitable performance loss (in terms of speed or accuracy) or to use a cloud computing infrastructure with an online API. The situation is a bit different when active-sensor designs are exploited: in that case, the identity of an individual is evaluated using not only the 2D image grabbed by a webcam but also analyzing a 3D map of the face. Such a system is more robust but way more expensive, and its distance range is limited by the projector power. In this paper, only monocular passive face sensor systems will be taken into account.

### 1.2. Framework

**Figure 1:**
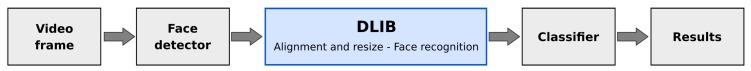
View of the former Dlib face recognition framework signal chain.

In a previous work [9] an open-source framework (Dlib [10]) for face-recognition has been identified and exhaustively tested. The framework, presented in Figure 1, consists of a selection of a face detector, an alignment procedure, an embedding oracle (feature extractor) and an identity classifier. The latter component can be implemented from scratch in the form of a shallow multi-layer perceptron (MLP) neural network (highest accuracy, short mandatory training phase) or with a simple distance metric (lower accuracy, insertion of identities in the database at runtime). Each classification algorithm is run on the features provided by a features extractor CNN (dlib-resnet-v1) [11] that is released as a part of the Dlib library in the form of a pre-trained model; in conjunction with the subsequent classifiers, this embedding network proved to be sufficiently discriminative.

Besides this, while on a PC the presence of a CUDA-compatible GPU permits a reasonable processing rate of 5–10 fps, on mobile hardware with an ARM CPU the average speed is in the order of roughly 0.5 fps (with Dlib compiled using ARM NEON [12] instructions), making mobile use impractical. Another macroscopic problem of this pre-trained model is that it has been created within the Dlib framework. As a consequence, further modifications, fine-tuning and research, as well as a simple conversion of the model represent an unnecessarily difficult burden [1].

### 1.3. Paper Outcome

In a previous work, we shared our findings on the distillation of dlib-resnet-v1 into a smaller model that could be implemented on mobile hardware for the recognition of tens of identities. Previous results were encouraging but were trained using a private dataset, composed of a mixture of the ones available online. The crucial point lies in the fact that we wanted to make a dataset as much as possible similar to the one used for the training of the teacher network. The present contribution, on the contrary, describes two feature extractors (the distilled models) obtained by distilling with a standard dataset only (CASIA): the first one can be directly inserted in the place of dlib-resnet-v1, while the second one provides a novel distilled network obtained including a spatial transformer component [13] in the structure; this not only removes the landmark detector and the face aligner (moving towards an end-to-end neural approach), but also allows to obtain a higher accuracy in the case of non-perfectly-frontal faces. Commonly used face detectors (Viola–Jones [14] and HOG [15]) are sensitive to pose and fail to detect most non-frontal faces. The use of newer face detection algorithms, typically CNN-based (SSD [16] and MMOD [6]), solves the aforementioned problem; however, the subsequent phase of landmark detection may provide wrong results, reducing the accuracy of the entire system. The described the Spatial Transformer Network (STN) boosted model correctly gives attention to the face part in a wide ROI and produces an aligned and tight crop of the face for the subsequent feature extractor, also for difficult poses. As far as we know, the distillation of an STN model is a novel procedure, fighting the idea that STN are difficult to train.

Differently from the previous work, this paper includes the LFW face verification test, which is a standard test procedure composed of a dataset and a testing protocol. This test is crucial to estimate the embedding skill of a neural model. In most research, the final model is trained on face recognition tasks that involve a closed set of individuals, just like any classification, neglecting the fact that in order to actually use this model it is necessary to expand the set. If a classifier is completely embedded into the model, the entire procedure is cumbersome. In this work, we emphasize the need for a modular procedure with a clear distinction of a feature extractor and a classifier, where the latter is a simple customizable structure. Furthermore, as far as we know, in this paper, a face recognition test procedure on an open set is formalized and described for the first time. We hope this will fill an empty spot in the field of the evaluation of the face recognition ability of the model.

This paper is organized as follows: the first section describes the framework, the distillation technique and the network design; the second section describes the testing methodologies, while the last one provides a discussion of the results.

## 2. Materials and Methods

### 2.1. Transfer Learning and Model Compression

When a model is trained to accomplish a task, it is convenient not to start from scratch (e.g., Gaussian or Xavier initialization) but to apply ‘transfer learning’, that is to copy as much as possible the weights of a previous well-trained network to the one that has to be trained. It has been demonstrated that starting in this way is generally more effective (not in any case, e.g., [17]) than starting with no description of knowledge at all (random initialization), even if the tasks of the two networks are different. One of the best starting points for computer vision tasks is the set of pre-trained weights obtained for the classification of the Imagenet dataset. It is important to note that in order to take advance of this sweet spot in the Loss-Parameters space the network configuration and structure have to be kept equal, possibly also wasting resources (e.g., replicating the number of channels of a grayscale image, or scaling the input image to fit the size of the first layer).

As reported in [18], applying concepts from the vast topic of model compression is the first step for reducing model complexity, and this result is obtained just by reducing the computational cost of each operation, without changing the structure of the model. The reduction of the time and memory complexity is instead a process that involves both structure simplification and a reduction in the number of parameters; the sweet spot is given by a reduced set of parameters and a smart choice for the data processing flow that maintains the same level of accuracy as the original network [1].

### 2.2. Model Distillation and Teacher–Student Approach

The first section highlighted the need for a complex structure to achieve the complex goal of face recognition. In fact, what requires complexity is the extraction of general characteristics from the provided samples (during the supervised learning process), rather than their actual representation. This means that when this knowledge has been inferred, it can be eventually represented by a simpler structure that can, in turn, be deployed to mobile hardware [18].

A recent and detailed survey on the general principles of distillation and model compression is presented in [19].

The form of compression [18,20,21,22] used in this work decorrelates the accuracy that a model achieves when performing a task from its learned weights: what is important to transfer (to distill) into a new model is the I/O relationship of the model itself, or the capacity to reveal the latent conditional distribution p(T|X) that relates the inputs *X* and the outputs *T*. This capacity is called ‘dark knowledge’ [21] and the act of transferring it from a slow but well-trained model (the teacher) to a student model is called ‘knowledge distillation’ [22].

The training set for the distillation process carried out as supervised learning is composed of the tuple (X,T), i.e., the input and the corresponding target. The distillation is carried out as a regression process, forcing the student network to provide the same descriptor generated by the teacher; in the case of an embedding network, this can be directly described in a distance metric framework, where a distance larger than the hypersphere radius of each cluster automatically flags bad learning. This motivates to choose as a loss metric the Euclidean Distance $L_{d}$ [18] calculated between the target feature vector *T* and the corresponding predicted descriptor *Y*.

The recent paper [23] proposes a peculiar knowledge distillation method composed of two different phases that explicitly takes into account smaller size and low-quality faces. In a complex training procedure, firstly the teacher network is frozen and a trainable structure of fully connected layers is attached to it. This model is then trained using a classification loss. The student model is distilled in a similar fashion of the previous “annealing based distillation” of Hinton. Unfortunately, this complex procedure leads to poor results in terms of the LFW face verification test.

The paper [24] presents a model for person re-identification, distilled from an ensemble of teacher models. Again a complex framework is exploited, in which a log-Euclidean distance is used as a loss function over sample similarity matrices. The framework automatically decides the reliability of each teacher in an adaptive fashion.

The paper [25] explores different techniques for using pre-digested information or in the paper called “privileged information”. In the paper, the term distillation is again used to denote the student training of output probability vectors, while the term “knowledge transfer” is used to denote a procedure that only slightly resembles our method: a mapping function is estimated that manipulates the features of the teacher adapting them for the student network.

Summarizing, the majority of the knowledge transfer methods based on distillation supervise the learning of intermediate features, or of output probability distribution (classification, soft-classification), eventually with the help of samples similarity-like matrixes. The only cases in which an output descriptor is somehow distilled [20,25] take into account just the adapted version of these features. In our work we designed a simple procedure for distillation in the metric framework that results in the training of a model completely different (and smaller) from the teacher, exploiting a different and smaller dataset composed of samples of low-quality images (image are reduced to a fourth). The testing of the distilled models is carried out on completely different identities (not only different images of the same id) unseen during the distillation, so the real generalization power of the model is tested. Making this entire training framework straightforward allows us to use distillation as an effective technique also for the initialization of a newer model, where training from scratch would require weeks of training.

### 2.3. Alignment Procedure and Spatial Transformer Network

Conventional network models, in general, do not have a high degree of spatial invariance. This makes the ROI cropped by a face detector not usable directly without a huge classification accuracy drop. If correctly realized, a face alignment procedure solves this problem by applying a spatial transform that brings face parts (eyes, mouth, nose, chin) on fixed points in the frame; the aforementioned procedure relies on a landmark detector (LD) in charge of searching for those landmarks within the frame. In the Dlib framework, the LD used is an implementation of the Kazemi-Sullivan algorithm [26] based on regression trees. Other approaches use local binary patterns [27] or a joint face detector/aligner structure based on SVM [28]. MTCNN [7] is one of the most effective CNN-based face-detector/landmark detectors and its recent implementation in Keras [29] increased its popularity. Research on multi-pose LD opened the way to 3D alignment: however, even if the most powerful methods (GAN [30] and symmetrization [31]) are optimal for restoration or entertainment purposes, 3D alignment did not show to provide significant advantages in terms of recognition accuracy over its 2D version [32].

A Spatial Transform layer [13] is a clever solution that has been introduced to provide spatial invariance to feature maps by applying a predefined spatial transformation on it; while stride and pool are fixed hyperparameters, STN transformation has parameters that are learned during the training of the entire model. The component that is responsible for the generation of suitable parameters is the so-called localizer, a shallow CNN which is responsible for the efficiency of the entire structure. A sampling grid is generated on-the-fly starting from the inferred transformation parameters and the gradients are calculated for the sampled points. When an STN is used as the input layer, an interesting effect happens: the network focuses on the portion of the input frame that it deems relevant for the task at hand. This is recognizable by observing the output image generated after the STN sampler. This fact can be used to localize a single object or a ROI within the frame or, like in this work, to localize a face in wide a crop (e.g., as provided by an uncertain face detector). In [33] an STN is used as well as the base of a neural face-detector STN, but with an important difference: the first stage is composed of a multi-task Region Proposal Network, which produces candidate ROI within the frame. Only in second place, the STN is used for the alignment of this candidate regions onto a canvas of predefined landmarks, whose positions represent some of the parameters to be learned. If the exploited transformation in an STN has at least four degrees of freedom (DoF) (e.g., it is a similarity transform), the byproduct of this method is a simple yet effective alignment of the face. In the influential [34] a shallow input STN (exploiting affine transform) is used as the input structure, and the following recognition model is simultaneously trained from scratch using a combination of loss functions.

In our work, we cascade an STN similar to the one above, with a different recognition model (the topic of the next section). The entire structure is then trained using distillation, following the teacher-student approach.

### 2.4. Contribution

#### 2.4.1. Network Architecture

The teacher Dlib network ’dlib-resnet-v1’ is based on a ResNet-34 structure [35] with few layers removed and the number of filters per layer reduced by half [10]: It has a 150 x 150-pixel input size, 29 convolutional layers and one fully-connected output layer for a total of roughly 6 M parameters. The network is provided pre-trained (for two weeks) on a dataset composed of roughly 3 M images. Due to its training procedure design, it is referred to as an embedding network because, for any given input image (an aligned face), the model provides a 128-dimensional features vector which virtually belongs to the embedding of that particular identity. During the training, a fixed distance margin is imposed between different identities meaning that all the possible images of a defined person would lie in a hyper-sphere of radius lower than the margin (0.6).

The student network design is crucial because, in principle, the computationally lightest model that allows us to obtain the performances of the teacher has to be defined. We can state the problem similar to the search for an ad-hoc optimal lossy compression for an average input distribution, evaluating a similarity metric.

Different CNNs based on the Densenet121 model [36] were designed searching for a structure with fewer weights than the original dlib model. This network design uses a combination of dense blocks, where features at different convolutional layers are concatenated, and transition blocks, where the features are processed and reduced to limit the pyramidal growth. Compared to Resnet [35] or Unet [37], this structure produces a stronger gradient flow and is computationally more efficient.

**Figure 2:**
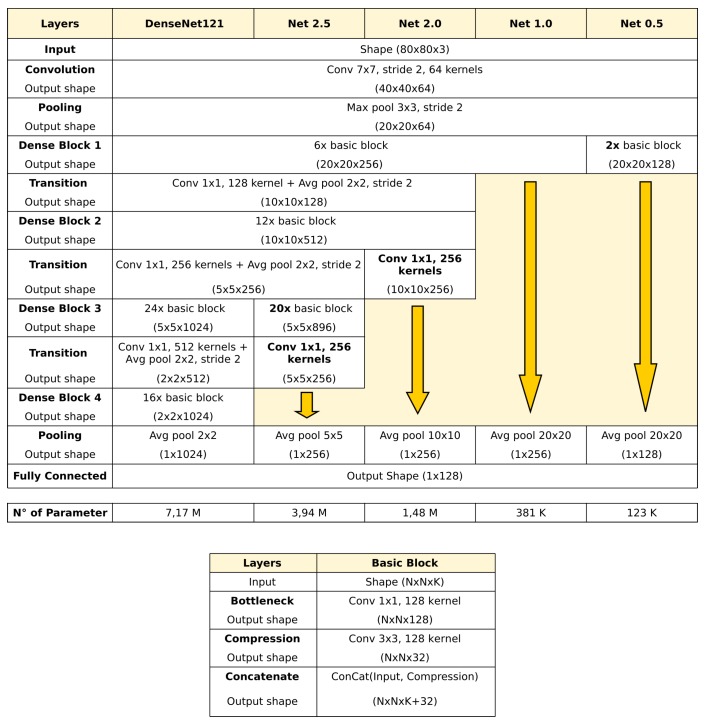
Schematic representation of the original Densenet121 model (first column) and our four different variants. In this work, Net 2.0 (3rd column) is chosen as our base recognition network.

**Figure 3:**
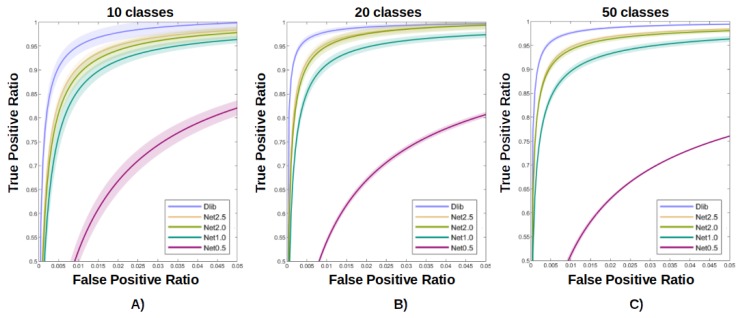
Performance evaluation of the different variants of the base distilled network [Net 0.5, Net 1.0, Net 2.0, Net 2.5] for the face recognition task in the case of 10 (**A**), 20 (**B**), 50 (**C**) classes. The procedure used was similar to the one described in Section 2.4.2. The performance over # parameters ratio is extremely competitive for Net 2.0 (1.48 Mparameters), while Net 2.5 (3.94 Mparameters) provides only a limited amount of performance gain for its number of parameters with respect to Net 2.0.

After training and testing four different variants [1,18], obtained cutting the Densenet at a different number of dense-transition blocks (also in the middle), we decided to choose the second biggest network (Net 2.0) as our base for the evolution of the network with the STN. Net 2.0 yields a reduction by a factor of 3.7 in size and by one order of magnitude in processing time (with HW accelerator), which is considered acceptable. The performance gain This can be seen in detail in Figure 2 and Figure 3.

**Figure 4:**
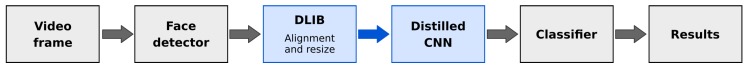
Signal chain of the hybrid framework composed by keeping the former face detector and alignment process; our distilled Convolutional Neural Networks (CNNs) block substitutes ‘dlib-resnet-v1’.

A strong reduction in computational complexity is achieved also by limiting the image input size at 80 × 80 pixels, thus forcing smaller faces (trough distillation) to be described by the same point computed with a frame four times larger (Figure 4).

**Figure 5:**
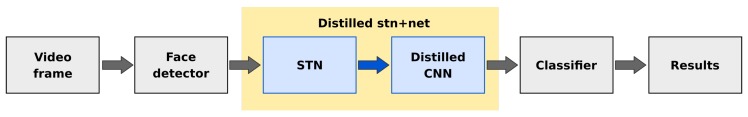
Signal chain of the novel hybrid framework composed by removing from the former the alignment procedure, which is substituted by the Spatial Transformer Network (STN) component in our ‘distilled stn+net’; the end-to-end structure encloses also our feature extractor network, in place of ‘dlib-resnet-v1’.

In order to cope with difficult poses and to enforce a better distillation, we modified the previously described network, adding an STN structure that acts as a neural face aligner, as shown in Figure 5.

**Figure 6:**
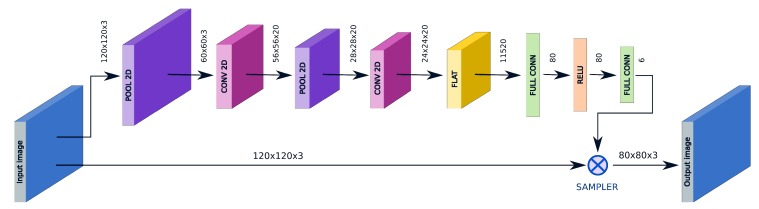
Structure of the STN component: an input image is processed by a shallow CNN-based localization network. The convolutional feature maps are then processed trough two fully connected layers that generate the six parameters of the affine transform. A grid generator (not represented) generates the corresponding sampling grid, that will be actually sampled by the sampler, producing an automatically aligned and cropped version of the input.

Like in [13], for the localization network we experimented with a shallow cascade of convolutional layers followed by a sequence of two fully connected layers whose output provides the six parameters of an affine transform. Differently from the previous case, the input size of the STN component is set to 120 × 120 pixels to help the localization network and provide a stronger free-data augmentation, but the final size of the transformed image is still 80 × 80; no modifications are needed in the recognition network. The structure detail is presented in Figure 6.

As far as we know, distillation on an STN based structure has never been attempted in the literature.

#### 2.4.2. Multiclass Open Set Problem

Intuitively, the problem that a face recognition network will solve is to correctly classify identities. In an example access control system, subjects belonging to the group of “friends” have to be recognized not only as members of that group, but in their specific identity too, in order to avoid authentication errors. Concurrently, for “unknowns” the access must not be granted.

To emulate this problem (and evaluate our models), a multi-class classifier has been designed starting from the features generated from each image: the training process consumes the features of friends only, resulting in an n-classifier for ”n-friends”, with n-outputs. During a test procedure, the classifier decides and we keep track of its decision, counting how many times a correct or a wrong choice has been made. In a closed set, the procedure is limited since all the possible cases, as well as all the possible individuals, can be evaluated. An example of this kind of classification is object recognition, in which a trainable oracle has to decide among a limited number of objects.

In the case of an open set, on the contrary, the cases to be considered are non-numerable. A common-sense way to tackle this problem is to estimate a confidence index related to the classifier decision. By adopting such an index it is possible to discriminate unknown subjects (for whom a classifier has not been trained), basing on the probably lower confidence of their identification. Since in most applications, a false-positive error is more dangerous than a false negative, the identification accuracy of known subjects can be increased granting access only to those with a high confidence index.

Defining the performance of a multiclass classifier depends on the scenario in which the classifier operates. In fact, some indexes or parameters which are usually adopted for a binary classifier can hardly be fostered in the case of a multi-class classifier.

More formally, we define a set of positive examples (Ni) belonging to the group of known identity (“friends”) that must be correctly classified (n-classes) and a set of negative samples (*F*), belonging to unknown individuals, that are used only to test the classifier, as no sample of this set have been seen during the classifier training. These latter samples, if correctly classified, represent the true-negative (TN), in respect of each class of known people and therefore they should not contribute in the evaluation of the global true-positive rate (TPR) like in the binary case. On the other hand, if they were incorrectly classified, they would represent false-positives (FP) for our system. Moreover, if a sample of a “friend” is erroneously classified as an ‘unknown’, this does not lead to an increase in the FP, but rather represents a false-negative (FN), whose impact on the evaluation of the classifier performance acts in a different way. Thus, we propose the following formulas for the calculation of the TPR and FPR in the case of multi-classification in an open-set scenario. A demonstration of these formulas is provided in Appendix A.

(1) $$TPR=\frac{\sum_{i=1}^{K}TP_{i}}{N}$$

(2) $$FPR=\frac{\sum_{i=1}^{K}FP_{i}}{K*F+(K-1)N}$$

where *K* is the number of classes used, *F* represents the total number of the negative samples (‘unknown’ or ‘others’ ID) and *N* is the total number of the positive sample (known ID or ‘friends’).

## 3. Distillation Experiments

### 3.1. Distillation Process

**Figure 7:**
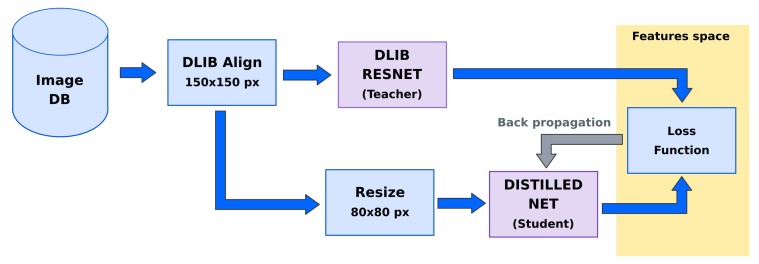
Proposed system with Dlib Resnet as teacher and Distilled network as student.

In this section, it will be described how the distillation takes place. Compared to the Dlib network, the two design choices that allowed for an extensive parameter reduction (5.58 M vs. 1.48 M) in the distilled network are the use of modern network design and the reduction of the image input size from 150 × 150 × 3 to 80 × 80 × 3 pixels. The computational complexity has been reduced maintaining a comparable recognition accuracy. In the following text, we will refer to this realization as ‘distilled net’. Besides the differences, this first distilled oracle can be used as a direct substitution in the former framework (Figure 7).

**Figure 8:**
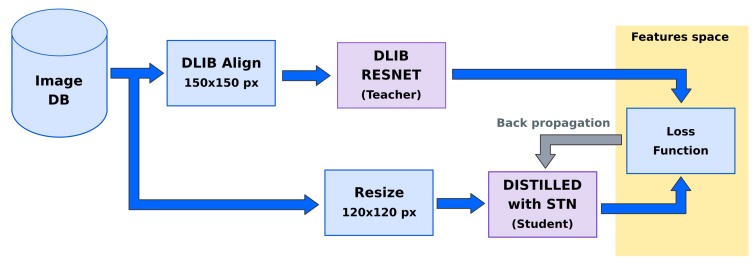
Proposed system with Dlib Resnet as teacher and Distilled network with STN as student.

Furthermore, in our second realization, which can be seen in Figure 8, an input STN structure is added to the model: the net effect of this change is in an improved recognition accuracy especially in a less constrained scenario. An STN with 120 × 120 × 3 pixel input and 0.93 M parameters is proposed; the overall network, which we will call ‘distilled stn+net’, uses 2.41 M parameters.

To train the two distilled networks we adopt the CASIA Web Face [38] dataset, composed of approximately 500 k images for 10.6 k identities, while the LFW [39] dataset is used for the subsequent tests; these two datasets have an overlap of 16 identities, which have been removed from the training dataset, in order to test the generalization capacity of the distilled model. Each dataset was “filtered" with Dlib’s HoG face detector: in this way, images with multiple faces were discarded. In the end, for each image, we have collected the corresponding features vector generated by the ‘dlib-resnet-v1’ model.

A set of (image,target) tuples is consumed during the training procedure, carried out forcing the student network to regress the target features vector for each image. For each RGB sample, the preprocessing step involves just a [0, 1] normalization and a per-channel shifting, while for each target vector the dataset average feature vector is subtracted. This procedure will simply change the origin of the 128-dimensional feature space.

In the case of the ‘distilled net’, all the color images have been aligned following the Dlib framework procedure and have been resized to 80 × 80 pixels. In contrast, for the ‘distilled stn+net’ no landmark detection and alignment was needed, and the dataset images have been only resized to 120 × 120 pixels.

For each distillation, we decided not to use any data-augmentation procedure because, in the described regression teacher-student approach, for each augmented sample we would have to generate the corresponding descriptor, inflating enormously the dataset dimension. In the case of ‘distilled stn+net’, again no data augmentation is enforced. During the training, the small fluctuations in the STN parameters (due to infinitesimal but nonzero gradient components) lead to a different image at the input of the recognition network, providing an effective data augmentation. At the same time, we have seen no signs of overfitting for the STN (that undergoes no augmentation).

The training of the nets continued for 100 epochs on batches of 128 images using Adam as the optimizer of choice. The supervised learning procedure evaluates the target error in terms of the Euclidean distance. The validation set consists of 1% of the train tuples, isolated at the start of each training.

### 3.2. Model Testing

The comparison and evaluation of the two distilled network, with respect to the former ‘dlib-resnet-v1’ is carried out using the LFW [39] dataset on two computer vision problems: face verification and multi-class face recognition in an open set. In order to make decisions, a form of classification has to be inevitably introduced. In this section, we are not only testing the models, but also the entire procedure that a potential user of the network has to fulfill (train of the ad-hoc classifier) in order to actually use the network itself. In fact, the features are just a mere representation of the identity, made invariant to lighting, pose and system conditions (within the input image). Training with the CASIA dataset and testing with LFW is a pretty well-standardized procedure and permits a robust and immediate comparison among methods; results on other datasets (e.g., Megaface [40]) are less widespread. In this work, we tested our solutions against 30 IDs, because the number of images available for each subject (in the testing dataset) was limited. In a previous work [1] however, we successfully tested our teacher network with a larger number of individuals, observing a limited performance drop.

#### 3.2.1. LFW Face Verification Test

The first problem is tackled by the use of the standard LFW test, consisting of a binary verification between pairs of images. The test represents a standard because in [39] the entire procedure to follow is described in [41] and then it is widely used in the Computer Vision community. A face verification procedure is the one used for automated airport check-in, where the same identity in the image grabbed by a camera has to appear also in the passport picture. In order to pass the face verification test, the algorithm under test has to correctly provide the answer to the question: “does the same identity appear in the two images provided?”. To do so, the LFW test provides 10 lists of 600 pairs of images (300 same ID, 300 different IDs). This is a binary test (2 classes: same ID, different ID), and a binary classifier has thus to be designed, since the output of our models is a feature vector, not a class. A classifier will produce a class response starting from the features. In this work, for the verification test, we opted for a Rocchio classifier, that exploits a simple distance metric; in such a classifier, only one trainable parameter is present, in the form of a distance threshold calculated ad-hoc on the validation dataset.

The procedure is split into two phases, called “View1” and “View2”: in the first phase the classification algorithm has to be designed (the design phase includes a testing of the classifier too) using a provided list of 3200 pairs, while in the second (10 × 600 pairs), the system is tested; the output of this second test, the real test, is processed to produce the accuracy value that can be communicated and compared to other solutions within the computer vision community. The purpose of the ten lists is to average these results. It has to be noted that the accuracy value estimated from the results does not depend only on the face recognition oracle itself, but on the entire framework used to process the images (e.g., the alignment procedure): in the case of ‘dlib-resnet-v1’ and ‘distilled net’, the aforementioned preprocessing procedure is the same and model-only performance differences emerge; for ‘distilled stn+net’, instead, changes in the figures involve also the alignment protocol.

Another proposed indicator consists of setting the maximum acceptable value of FPR and then evaluating the resulting TPR over the ROC curve, obtained by varying the threshold. Running the same test utilizing the ten lists provided by the protocol, it is possible to calculate the average and the standard deviation for each point.

#### 3.2.2. Multi Class Face Recognition in an Open Set

The objective of the second test is to evaluate the clustering ability of the embedding models, crucial for reliable recognition. During this test, the system has to recognize people that it knows (labeling the correct name) against images of not only the known ID (the so-called “friends") but also taken from random identities (the “unknowns”). In order to simulate the scenario of open-set in the standard LFW dataset an amount of identity is taken to form the group of “friends” and the remaining IDs compose the unknown set. Note that LFW has no overlap with the CASIA dataset that is used for the distillation of our features extractor model. As described in Section 2.4.2, a multiclass classifier is needed for face recognition on an open set. Following the work presented in [9,18], we adopted a shallow Multi-Layer Perceptron (MLP) formed by three fully connected layers: the first two consists in 100 neurons, while the number of outputs in the last one is the number of classes to recognize. The intermediate nonlinearity used is a ReLU, while for the final nonlinearity we opted for the Softmax activation function. In order to distinguish a subject that does not belong to known classes (’unknown’), we used the normalized distance as confidence index, for which the logit values are compared, according to Equation (3).

(3) $$C=\frac{d_{1}-d_{2}}{d_{1}-d_{n}},$$

where $d_{1}$, $d_{2}$ and $d_{n}$ are respectively the largest, the second-largest and the smallest value of the output layers.

The final decision is taken not only by observing the class of highest probability, but also the confidence value, calculated with Equation (3). For each classifier, we studied the effect of both a different number of classes and a variable number of samples provided to the model during the training. Table 1 summarizes the testing conditions. We set the number of classes (the output of the classifier) and we trained the model using 2, 5, or 15 samples for each identity to recognize. Only these samples are seen during the learning. During the testing, the number of images for each known ID is kept constant to 10. To test how well the classifier rejects unknown subjects, other samples have to be added (open-set problem).

To do so, a number of images of unknown identities equal to the number of image friends are used, randomly choosing from all LFW IDs who are not used as a friends. Working in this way no bias is triggered during the procedure. Details for all the explored cases are given in Table 1.

Following the procedure described in Section 2.4.2, we calculated the TPR and the FPR as a function of the estimated confidence *C* for a multi-class problem and we plotted the ROC curve of the classifier using Equations (4) and (5):

(4) $$TPR=\frac{\sum_{i=1}^{K}TP_{i}}{N}$$

(5) $$FPR=\frac{\sum_{i=1}^{K}FP_{i}}{K*F+(K-1)N},$$

where TP is the number of correctly classified samples (with *C* above the selected threshold of confIDence) and *N* is the number of known samples provIDed during the test; FP is the number of misclassified samples (the number of known people whose identity has been misclassified plus the number of the unknown people which are classified with a confidence index above the threshold, i.e., faces that have been erroneously classified as a known person) and *F* is the number of all the unknown samples.

Using the LFW dataset, only 30 identities have at least 30 images each: according to this limit, the training of MLP was carried out using only 2, 5 or 15 images for each subject, reserving five images to the verification (early stopping in training) and 10 for the test. The remaining 10 samples of each known face are used for the test, while 10 × Nc images of other identities are enrolled to form the unknown people corpus. The number of unknown samples is chosen in order to balance the testing set: the entire procedure is repeated ten times for different individuals, in a cross-validation approach. The results of the various tests, at different thresholds of confidence, were represented in the ROC plane highlighting the area that contains 99% of the results and tracing the average ROC curve described by these values.

### 3.3. Hardware Implementation

The distilled network has been tested on a Single Board Computer (Odroid XU-4); the inference time of ‘dlib-resnet-v1’ (using the CPU, compiling Dlib with the Arm-Neon [12] flag) was compared with the distilled network using TensorFlow Lite [42] (CPU approach) and a hardware accelerator such as the Intel Movidius Neural Compute Stick (NCS) [43]. The mean inference time for Dlib is 816 ms, while for the ‘distilled net’ 195 ms are needed for its TensorFlowLite porting and only 67 ms are needed if the hardware accelerator is used, providing a speed gain of one order of magnitude, keeping the same accuracy.

TensorFlowLite and the Intel embedded converter are able to synthesize standard layers only, such as dense, convolutional, activation and so on. Unfortunately, the conversion of the STN boosted network (‘distilled stn+net’) is currently impossible due to the presence of the unconventional sampling layer. We hope that in a future version of the tools this conversion can be done.

## 4. Results and Discussion

### 4.1. LFW Verification Test

The first test was conducted on the original Dlib network and on the two proposed distilled networks using LFW dataset in order to evaluate their verification ability.

Table 2 summarizes the average results obtained from the 10 tests proposed by the LFW test: the accuracy was calculated following the protocol defined by LFW, while TPR value with desired FPR constrain was calculated as explained in Section 3.2.

The table shows that the distillation of the dark knowledge was successful: The accuracy of the two distilled models is comparable to the one of the teacher. Another interesting view on the verification test result is obtained choosing a threshold on the maximum acceptable FPR and reading on the ROC curve the corresponding value of TPR.The solution ‘distilled stn+net’ provides a TPR value even higher than the one of ‘distilled net’.

### 4.2. Recognition Test

**Figure 10:**
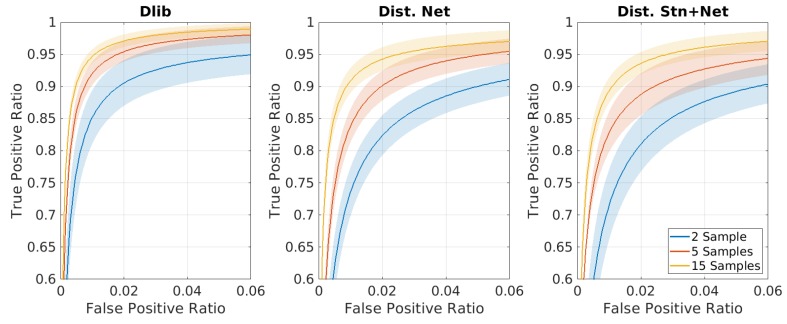
Average ROC curves estimated from a ten-fold cross-validation procedure on multiclass classifier. Each classifier is trained using a fixed amount of classes (30) and a varying number of training sample, using the features generated by ’dlib-resnet-v1’, ‘distilled-net’, ‘distilled stn+net’. Note that the graphs are highly zoomed portions of the entire curve.

**Figure 11:**
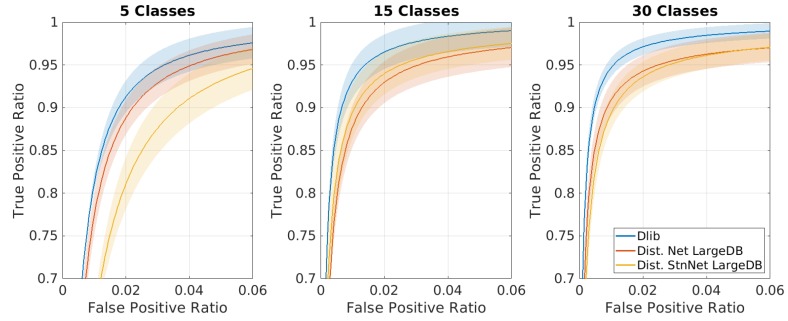
Average ROC curves estimated from a ten-fold cross-validation procedure on multiclass classifier. Each classifier is trained using a fixed amount of samples (15) and a varying number of classes, using the features generated by ’dlib-resnet-v1’, ’distilled-net’, ’distilled stn+net’. Note that the graphs are highly zoomed portions of the entire curve.

**Figure 12:**
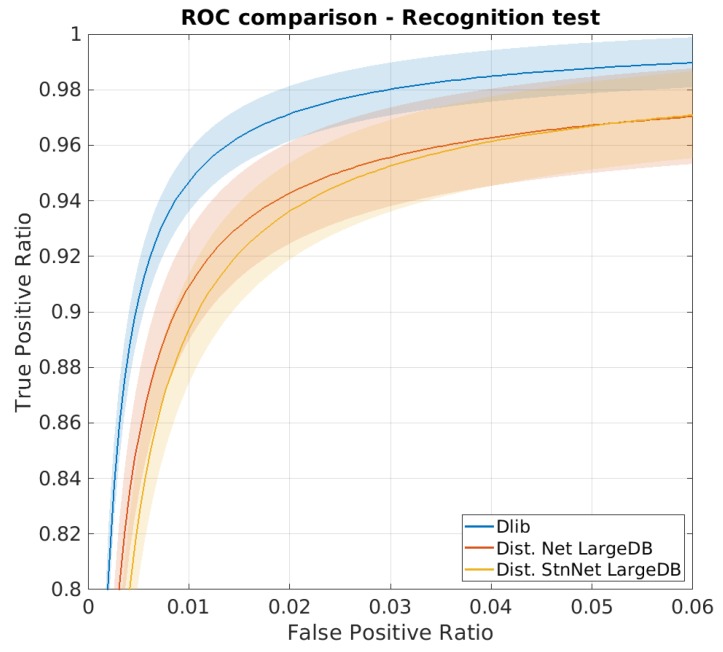
ROC curve comparison for the best case (30-class classifier trained with 15 samples per ID). Note that the graphs are highly zoomed portions of the entire curve.

The second test aims to compare the performance of the networks considering the problem of face recognition in an open set. In the following Figure 10, Figure 11 and Figure 12 the shadowed regions represent the areas that cover 99% of the results of the 10 tests, while the bold line represents the mean ROC curve for the former and the two ’distilled net’ and ’distilled stn+net’.

In Figure 10 many different ROC curves (the result of different classifiers), are produced imposing a varying limit on the number of samples used during the training. As described in the previous section, this test has been performed fixing the number of classes and then using the 30-class classifier only. It should be noted that even with the training of only two images per ID, it is possible to recognize a person in the wild with acceptable accuracy. This setup is particularly interesting e.g., for the automatic checking of suspect subjects of whom only a few photos are available.

In Figure 11, similar tests were proposed by fixing instead of the number of training samples to 15 and varying the number of classes (of known subjects) among 5, 15 and 30. Up to a certain limit, the entire framework is invariant to the class number, allowing for the best performances when the ‘known person’ database is composed of a few dozen identities.

For clarity, we have summarized the two results in Figure 12, comparing the ROC of the networks under test with the teacher network in the case of optimal parameters (30-class classifier trained with 15 samples per ID).

### 4.3. STN Analysis: Co-Adaptation and Difficult Poses

**Figure 13:**
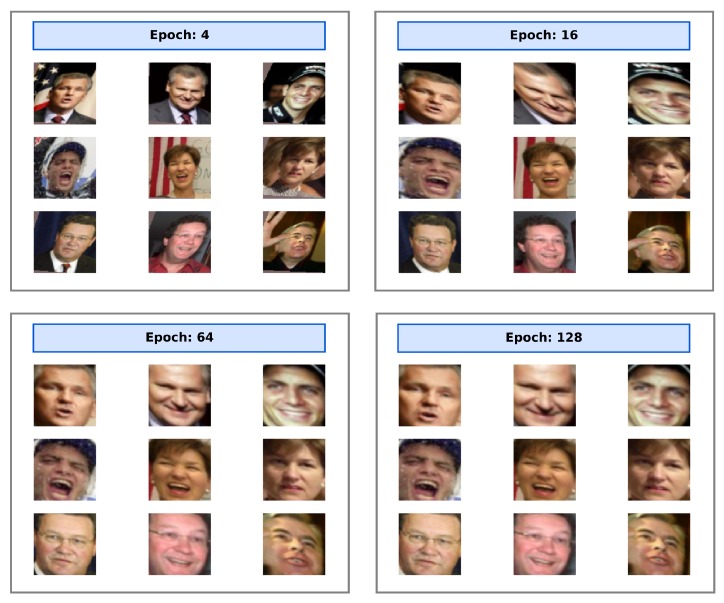
This figure shows the evolution of the STN output during the training of ‘distilled net+stn’. In less than 10 epochs, the STN correctly localizes the faces, while in 30 epochs the STN correctly learns to localize and align images for the recognition network.

The STN and the face recognition network are used in tandem after a common training phase. The only feature maps shared between the two is the STN output image which lies in the standard RGB image space. Analyzing this output image provides insight into the training of the entire model. At the end of each epoch, a callback launches the test, and for nine test samples, the output of the STN component is extracted and saved. Since this image is a mere feature map, we can analyze the co-adaptation between the STN and the recognition components and evaluate how the alignment skills are learned after each epoch. In Figure 13 the output of the STN aligner is reported for nine people after 4, 16, 64 and 128 training epochs.

We can observe that typically in ten epochs the STN component learned to isolate a face within the frame and found the best way to minimize the Euclidean distance loss function. Interestingly, the network automatically decided that the best possible alignment procedure (which minimize at most the loss function) consists of rotating the face by a few tens of degrees, in order to occupy the largest possible area, thus removing part of the background remaining around the hair and chin. It is reasonable that this behavior is forced also by the downsampling of the input image operated by the sampler in the STN.

**Figure 14:**
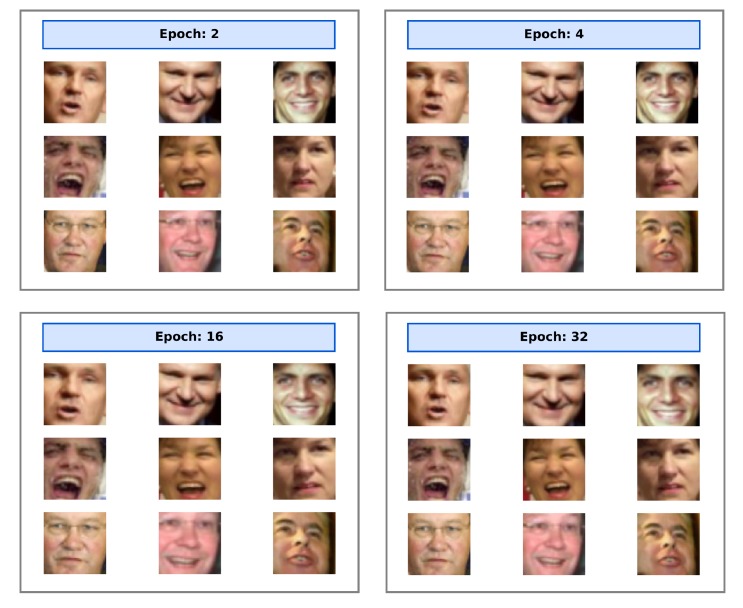
Differently from the previous case, this figure shows the evolution of the STN output for the training of the STN component only, providing a pre-trained ‘distilled net’ as the embedding model. In just one epoch, the STN learns to align each face putting the eyes horizontally, and emulating the crop factor of the former Dlib face aligner.

In Figure 14 a similar experiment has been carried out using a pre-trained and frozen distilled net, in which the STN was the only trainable component: in the processed face the eyes are aligned to the horizon; the STN learned in just one epoch to emulate the Dlib alignment procedure, localizing and aligning the face.

**Figure 15:**
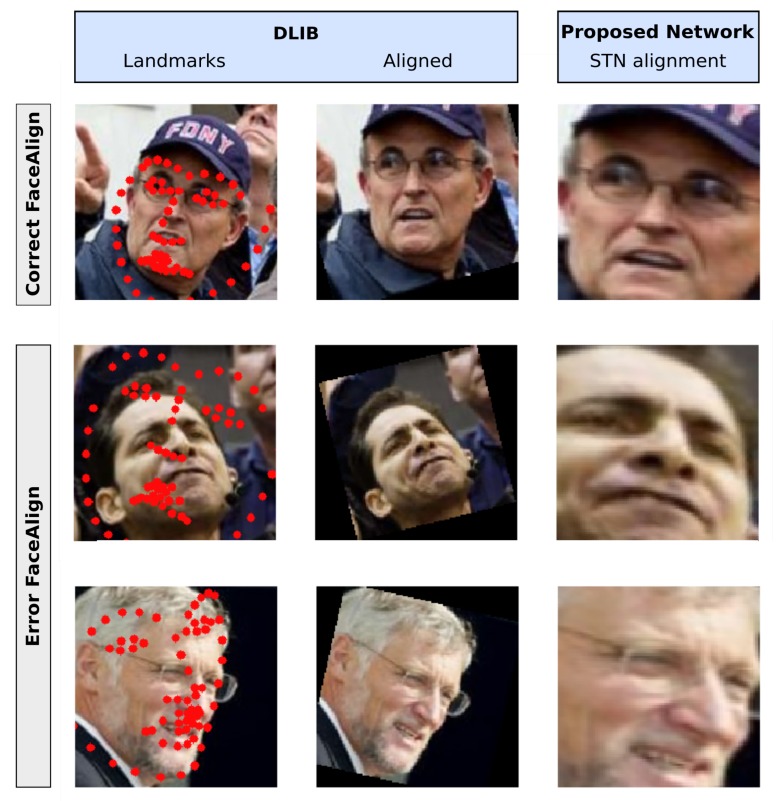
In this figure different alignments are compared for difficult face poses. In the first column, the landmark found with the dlib shape predictor are shown; in all the samples the error is heavily present. Only the face alignment procedure carried out on the first sample generates a correct recognition (a descriptor in the Euclidean space close to the centroid of its identity). The ‘distilled stn+net’ model is capable of correctly localizing and aligning the face, just like in any other pose.

The results presented till now take into account only the samples that can be actually analyzed by the Dlib framework, e.g., the ones that have been selected as faces by the face detector. The real advantage of using the STN distilled network emerges when difficult poses are recorded in the frame. In order to evaluate this aspect, we selected the samples from the LFW dataset where no faces are found (for a deficiency of the face detector). If a landmark detection is carried out on these frames, the subsequent alignment will produce images for which dlib-resnet-v1 cannot produce meaningful features. In Figure 15 we compare the alignment of the Dlib algorithm with the alignment obtained with our proposed model: the STN is, in any case, able to give attention to the face and to align it in a manner that makes the subsequent model able to verify the identity (the points in the hyperspace are closer than the threshold used for the binary ‘same–different’ verification test). We point out that the shown results are carried out on test samples, which the network had never seen during the training phase.

### 4.4. Distillation Strategy as a “Transfer Learning” for the New Model

**Figure 16:**
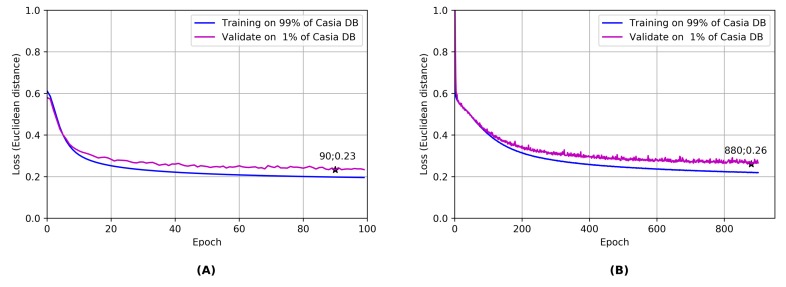
Two distillation trainings are compared: in panel (**A**), the ’full’ learning is exploited, providing the best results. In panel (**B**), a different strategy is used: for each training batch a different identity is extracted and only two random images are given to the network. This procedure is way faster than the previous one (1000 epochs in 6–8 hours vs 100 epochs in 15 hours) but obviously a bit less performing. During the distillation, what counts is the number of identities (cluster centroid), rather than the number of samples for each centroid.

Two different distillation training strategies have been followed: the first one enrolls the entire Casia dataset blindly, while the second exploits a predefined sample presentation structure in each training batch; in the second case, we fixed the number of different IDs for each batch to 64 with two samples for each ID. In this manner, even if the number of samples per batch remained constant in the two cases (128), each epoch lasted more than 10 times less, allowing to train in half the time, for 1000 epochs. In Figure 16 the two-loss evolutions are compared: The resulting accuracy is highly comparable, highlighting that for a correct distillation it is crucial to have different cluster centroids in the sample space.

From the graphs a second observation can be drawn: distillation can be enforced as a fast initial training technique for the training of the new network, as a “transfer learning methodology” for newer networks, if the newer model under investigation has to answer to the same type of question.

## 5. Conclusions

In this work, we described two face recognition models that can be implemented on low-cost hardware, in the form of a face recognition sensor module. The key procedure exploited in this work is knowledge distillation, used to extract the dark knowledge of a dlib-resnet-v1 network in a teacher-student framework. Each distillation has been obtained in a simple metric framework, essential if distillation is used as an initialization technique. In this sense, a relatively fast distillation can be used as a “transfer learning” phase between different models. One model is a direct substitute of the original network, that can be then used without adaption layers; our second realization embraces instead of an end-to-end approach that permits to remove the separate alignment procedure. The second model proved to be definitely more robust in the case of difficult poses. To the best of our knowledge, a distillation of such a structure for face recognition has never been attempted. A well-acknowledged training and testing protocol has been exploited to evaluate the performances of each realization, in the form of the LFW face verification test and a novel face recognition in an open scenario test description. The outcome of this problem description is a procedure for unknown ID rejection that exploits a confidence measure and thus minimizes the false-positive error rate.

## Figures and Tables

**Figure 9:**
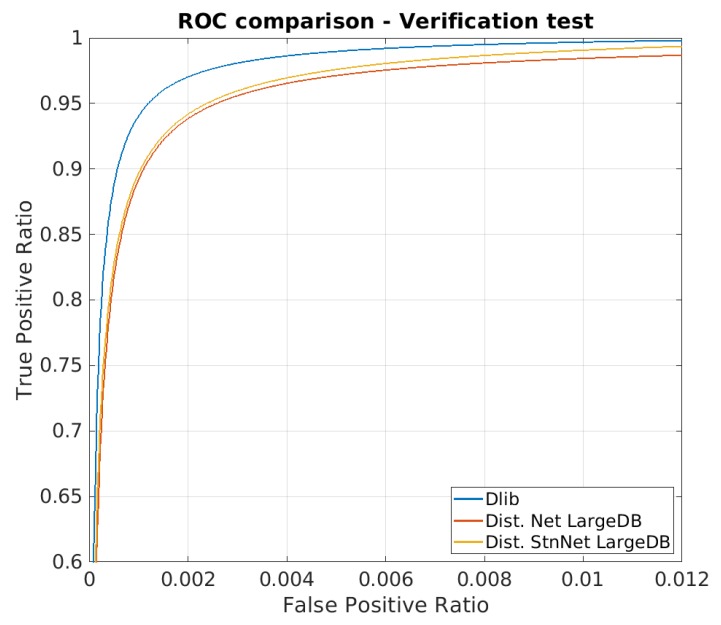
ROC curve for the LFW face verification test. Note that the graphs are highly zoomed portions of the entire curve.

**Table 1:** Performances in face recognition are evaluated for different classifiers trained with a different amount of outputs (classes). This table summarize the number of samples used in each case. A limited number of individuals (5, 15 or 30) is extracted from the dataset: these are the identities we want to recognize. During the training (and the validation), only known IDs samples are provided. Note that the desired number of training samples (2, 5 or 15) is kept constant for each class. This choice allows for a perfect balance between each class, giving no a priori information through sample distribution. During the testing, all the other identities outside of the closed set of known IDs can potentially provide unknown samples. The classifier is tested against the unknown IDs rejection providing a fixed number of images that is equal between the known and unknown individuals. Again, no predilection on a particular class or on the “unknown” is inferred.

CLASSES	# TRAIN Imgs (Known IDs Only)	# VALIDATION Imgs (Known IDs Only)	# TESTING Imgs (Known IDs)	# TESTING Imgs Unknown IDs
5	5 × (2 or 5 or 15)	5 × 5	5 × 10 = 50	50
15	15 × (2 or 5 or 15)	15 × 5	15 × 10 = 150	150
30	30 × (2 or 5 or 15)	30 × 5	30 × 10 = 300	300

**Table 2:** Comparative results of the LFW identity verification test. Each row provides the figure for each model, the former ‘dlib resnet-v1’ and our two distilled model, ‘distilled net’ and ‘distilled net+stn’. The first column shows the resulting accuracy, while the last two columns provide the TPR value on the ROC curve (Figure 9) corresponding to an imposed FPR value of choice.

Network	Accuracy	TPR @ FPR=1%	TPR @ FPR=0.1%
dlib-resnet-v1	0.9918 ± 0.0033	0.9923 ± 0.0049	0.9344 ± 0.1365
Distilled net	0.9852 ± 0.0050	0.9819 ± 0.0106	0.8931 ± 0.1051
Distilled stn+net	0.9852 ± 0.0058	0.9908 ± 0.0137	0.9067 ± 0.1241

## Appendix A

### Appendix A.1

Defining the performance of a multiclass classifier is not trivial and essentially depends on the scenario in which the classifier operates. In fact, some indexes or parameters which are usually adopted to define the performances of a binary classifier are not adequately suited to the case of a multiclass classifier.

One performance representation commonly used in binary classifiers is the ROC (receiver operating characteristic) curve. This curve represents the True Positive Rate (TPR) vs. the False Positive Rate (FPR), as achieved by the classifier at various threshold settings.

Such parameters in a binary classifier are defined as follows in Equations (A1) and (A2):

(A1) $$TPR=\frac{TP}{N}$$

(A2) $$FPR=\frac{FP}{F}$$

where *N* is the number of positive samples and *F* is the number of negative samples used to test the classifier. TP (True positive) is the number of positive samples correctly classified and FP (False Positive) is the number of negative samples erroneously classified as true. For completeness we usually define False Negative (FN=N−TP) the number of positive cases erroneously classified as negative and True Negative (TN=F−FP) the number of negative cases correctly classified.

The application of similar parameters in a multiclass classifier depends on the correct identification of the scenario. Suppose that the task is to classify an individual among a finite number *K* of classes knowing that the individual belongs to one and just one of these classes. It is possible to define the true positive rate for each class *i*, $TPR_{i}$, as:

(A3) $$TPR_{i}=\frac{TP_{i}}{N_{i}}$$

where $N_{i}$ is the number of samples, belonging to class *i*, submitted to the classifier, during the test, and $TP_{i}$ is the number of these samples correctly classified.

Now it is possible to extend the concept of True Positive Rate to the entire classifier, simply by averaging all the values of $TPR_{i}$ on all the classes, better if using a weighted average based on the number of samples submitted to the classifier for each class:

(A4) $$TPR=\sum_{i=1}^{K}\alpha_{i}*TPR_{i}$$

where $\alpha_{i}$ must be constrained: it must be proportional to the number of samples $N_{i}$ and $\sum \alpha_{i}=1$, thus:

(A5) $$\alpha_{i}=\frac{N_{i}}{\sum_{i=1}^{K}N_{i}}$$

Substituting Equations (A3) and (A5) in Equation (A4) and simplifying we obtain:

(A6) $$TPR=\frac{\sum_{i=1}^{K}TP_{i}}{\sum_{i=1}^{K}N_{i}}=\frac{\sum_{i=1}^{K}TP_{i}}{N}$$

where $N=\sum N_{i}$ is the number of samples provided to the classifier during the test.

Operating in the same way it is possible to evaluate the FPR:

(A7) $$FPR_{i}=\frac{FP_{i}}{N-N_{i}}$$

where $N-N_{i}$ is the number of samples belonging to classes other than *i* and $FP_{i}$ is the number of these samples erroneously classified. To define a global FPR value it is possible to proceed, like before adopting a weighted average:

(A8) $$FPR=\sum_{i=1}^{K}\beta_{i}*FPR_{i}$$

The constraints to the weights $\beta_{i}$ are $\beta_{i}\propto \left(N-N_{i}\right)$ and $\sum \beta_{i}=1$ thus:

(A9) $$\beta_{i}=\frac{N-N_{i}}{\sum_{i=1}^{K}\left(N-N_{i}\right)}$$

Substituting Equations (A7) and (A9) in Equation (A8):

(A10) $$FPR=\frac{\sum_{i=1}^{K}FP_{i}}{\sum_{i=1}^{K}\left(N-N_{i}\right)}=\frac{\sum_{i=1}^{K}FP_{i}}{(K-1)N}$$

However, the scenario we want to take into consideration in this article is slightly different from the one just mentioned. In our case there are both a number of positive examples ($N_{i}$) relating to the known subjects that must be correctly classified into a proper class, and a set of negative samples (*F*), belonging to unknown individuals, that have never been seen before by the classifier. These latter samples, if correctly classified, are TN (True Negative) with respect to each class of known people and therefore they should not contribute to the computation of the global TPR as in the previous case. On the other hand, if they are incorrectly classified, they represent false positives for our system.

On the other hand, an individual belonging to the positive samples who is erroneously classified as an unknown subject does not lead to an increase in the FP; rather, it represents a FN, whose impact on the evaluation of the classifier performance is very different.

Thus, while Equation (A6) for the calculation of the TPR can remain unchanged, the calculation of the FPR must be applied to all the cases in which an individual is attributed to the wrong class, considering as target all possible class but the one composed by unknown individuals. Thus Equation (A7) should be reviewed considering that for each class the negative samples come both from samples of the other classes (N−Ni) and from the ones which belong to unknown subjects (*F*).

(A11) $$FPR_{i}=\frac{FP_{i}}{F+N-N_{i}}$$

Thus the weights adopted in Equation A8 to evaluate the weighted average should be revised as follows:

(A12) $$\beta_{i}=\frac{F+N-N_{i}}{\sum_{i=1}^{K}\left(F+N-N_{i}\right)}=\frac{F+N-N_{i}}{K(F+N)-N}$$

Thus:

(A13) $$FPR=\frac{\sum_{i=1}^{K}FP_{i}}{K*F+(K-1)N}$$
